# Influence of Magnetorheological Finishing on Surface Topography and Functional Performance of Shoulder Joint Cap Surface

**DOI:** 10.3390/ma18143397

**Published:** 2025-07-20

**Authors:** Manpreet Singh, Gagandeep Singh, Riyad Abu-Malouh, Sumika Chauhan, Govind Vashishtha

**Affiliations:** 1 Department of Mechanical Engineering, Baba Farid College of Engineering and Technology, Bathinda 151002, Punjab, India; drmanpreetsinghbfgi@gmail.com (M.S.); gagan.1804@gmail.com (G.S.); 2 Applied Science Research Centre, Applied Science Private University, Amman 11931, Jordan; 3 Department of Mechanical Engineering, Chitkara University Institute of Engineering and Technology, Chitkara University, Rajpura 140401, Punjab, India; 4 Department of Mechanical and Industrial Engineering, Applied Science Private University, Amman 11931, Jordan; riad_abumlwah@asu.edu.jo; 5 Faculty of Geoengineering, Mining and Geology, Wroclaw University of Science and Technology, Na Grobli 15, 50-421 Wroclaw, Poland; sumi.chauhan2@gmail.com; 6 Division of Research and Development, Lovely Professional University, Phagwara 144411, Punjab, India; 7 Department of Mechanical Engineering, Graphic Era Deemed to be University, Dehradun 248002, Uttarakhand, India

**Keywords:** magnetorheological finishing, shoulder joint cap, surface roughness, wear resistance, biomedical implants, biocompatibility

## Abstract

The surface quality of biomedical implants, such as shoulder joint caps, plays a critical role in their performance, longevity, and biocompatibility. Most biomedical shoulder joints fail to reach their optimal functionality when finished through conventional techniques like grinding and lapping due to their inability to achieve nanometer-grade smoothness, which results in greater wear and friction along with potential failure. The advanced magnetorheological finishing (MRF) approach provides enhanced surface quality through specific dimensional control material removal. This research evaluates how MRF treatment affects the surface roughness performance and microhardness properties and wear resistance behavior of cobalt–chromium alloy shoulder joint caps which have biocompatible qualities. The study implements a magnetorheological finishing system built with an electromagnetic tool to achieve the surface roughness improvements from 0.35 µm to 0.03 µm. The microhardness measurements show that MRF applications lead to a rise from HV 510 to HV 560 which boosts the wear protection of samples. After MRF finishing, the coefficient of friction demonstrates a decrease from 0.12 to 0.06 which proves improved tribological properties of these implants. The results show that MRF technology delivers superior benefits for biomedical use as it extends implant life span and decreases medical complications leading to better patient health outcomes. The purposeful evaluation of finishing techniques and their effects on implant functionality demonstrates MRF is an advanced technology for upcoming orthopedic implants while yielding high precision and enhanced durability and functional output.

## 1. Introduction

As a body’s most advanced articulating joint the shoulder requires specialized prosthetic replacements with strength under mechanical stress, safe chemical substance interaction, and stable constructive properties [1,2]. Total shoulder arthroplasty depends on shoulder joint caps to function as articulating surfaces during replacements because they help restore mobility to patients who have degenerative diseases, trauma-related injuries, and congenital handicaps. Shoulder joint caps come from materials such as cobalt–chromium alloys, titanium alloys, and medical-grade stainless steel because these materials show excellent strength alongside corrosion resistance properties and biocompatibility [3]. The materials used for these elements need highly detailed finishing processes to reach friction levels that prevent wear while ensuring exceptional bone tissue integration. The shoulder joint cap’s performance duration is drastically affected by its finishing quality because poor surface quality creates rapid material reduction resulting in metal part generation and implant failure together with inflammatory tissue responses [4,5,6,7].

According to recent market reports, the global orthopedic implant market was valued at USD 50.2 billion in 2023 and is projected to reach approximately USD 75.1 billion by 2030, growing at a compound annual growth rate (CAGR) of 6.5% [8]. This growth is driven by an aging population, increasing prevalence of orthopedic disorders, and the need for long-lasting, high-performance implants. Surface finishing processes like magnetorheological finishing (MRF) play a critical role in enhancing implant durability and biocompatibility, thereby reducing the risk of revision surgeries and improving patient outcomes. Orthopedic implant manufacturing traditionally uses grinding and lapping, conventional polishing techniques which fail to reach nano-scale smoothness especially when working with complex geometries such as joint caps [9]. The use of abrasive particles on a wheel serves as a material removal method which produces dimensional accuracy together with surface quality outcomes. Traditional finishing methods using this technique result in several severe drawbacks, which include heat-generated damage, additional stress formation, and dimensional changes in the material [10,11,12,13,14,15]. Lapping represents a well-known finishing method which employs abrasive particles in a liquid solution to enhance the finish quality between two surfaces. Surface smoothness from lapping is superior, but the process lacks any control over the material removal rate, which results in non-uniform finishes [12,13,14]. The process of conventional mechanical polishing uses rotating polishing pads alongside abrasive slurries yet faces restrictions in delivering consistent results over intricate shapes. The development of advanced finishing techniques became necessary after scientists sought methods for better control of material removal and superior surface quality production [15]. Magnetic Abrasive Finishing (MAF) together with Rotational Magnetorheological Abrasive Finishing (RMAF) and Ball-End Magnetorheological Finishing (BEMRF) represent advanced finishing procedures which serve as superior substation to classic finishing approaches [16,17,18]. A magnetic field within MAF controls abrasive particles’ movement for accurate material removal while avoiding the production of residual stresses. The technique delivers excellent results when finishing locations are hard to access and have complex shapes [19]. The extension of MAF technology into RMAF uses a spinning workpiece together with regulated abrasive motion to improve finishing precision and uniformity. Strong magnetic fields and precise abrasive particle distribution are required to make MAF and RMAF effective finishing methods. By implementing the ball-ended tool during the BEMRF process, engineering scientists have developed a solution which addresses these restrictions by creating focused finishing effects [20,21,22,23,24,25,26]. The new technique enables the precise manipulation of finishing operations to effectively work on orthopedic implant components like shoulder joint caps. The MRF process acts as the central element of this study because it delivers unsurpassed surface finishing efficacy through magnetic manipulation of small abrasive particles to create surfaces at nanometer precision. MRF gives operators real-time access to control finishing parameters that lead to uniform material removal from complex geometries [27]. The MRF processing system delivers controlled finishing results and ultra-smooth surfaces with minimal material loss, which qualifies it as the leading technique for biomedical implant surfaces. MRF for biomedical applications has undergone thorough research proving the technology can achieve surface roughness values of 0.03 μm thereby outperforming traditional methods by a wide margin. The joints’ functionality benefits from an ultra-smooth finish on the surface, which improves lubrication properties, decreases joint friction, and ensures simple motion operation [28,29,30,31,32,33,34,35,36,37,38,39,40,41,42,43]. MRF shows flexibility because it enables customized process optimization through which users can achieve consistent finishing performance on different implant materials [29]. The bacterial adhesion on SS-BP-MPC composite surfaces was reduced by 2.5-fold compared to pristine stainless steel, primarily due to the combined effect of lower surface roughness and improved hydrophilicity. This highlights the importance of nano-smooth surfaces in suppressing microbial colonization on biomedical implants [30]. To meet expanding high-performance biomedical implant standards, the medical community needs to implement advanced finishing methods like MRF because it boosts implant reliability, stops revision surgeries, and delivers superior patient results. This research undertakes an evaluation of MRF as an effective surface finishing method for shoulder joint caps by investigating its capacity to improve surface properties including smoothness, wear resistance, and biocompatibility. A systematic evaluation of MRF finishing technology depends on a process parameter analysis including tool rotation speed, workpiece movement, magnetic field intensity, and abrasive concentration to prove MRF’s position as an optimal finishing solution for orthopedic implants. Research findings generated in this work will lead to crucial insights about improving finishing procedures for medical applications thus enabling MRF’s mainstream acceptance in implant production.

The function of shoulder joint caps as well as their service life primarily depends on their finishing quality. Traditional finishing approaches used for implant surface improvement fail to deliver enough precision needed for biomedical applications despite their benefits in surface enhancement [17,18,19,20]. MRF stands out as the superior finishing technique due to its ability to adapt and deliver advanced precision and high-quality finishing capabilities among all existing methods of advanced finishing, including grinding, lapping, MAF, RMAF, and BEMRF. MRF technology enables orthopedic implant makers to produce shoulder joint caps which deliver superior medical-quality components that improve joint movement alongside patient health benefits.

## 2. Materials and Methods

**Figure 1:**
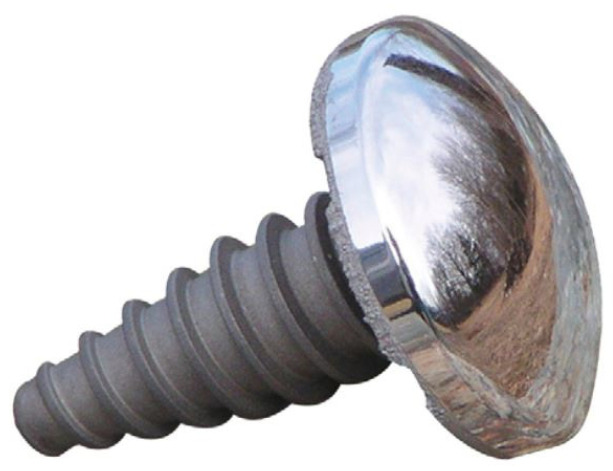
Cobalt–chromium (Co-Cr) alloy-material shoulder joint cap used as a workpiece during MR finishing.

**Figure 2:**
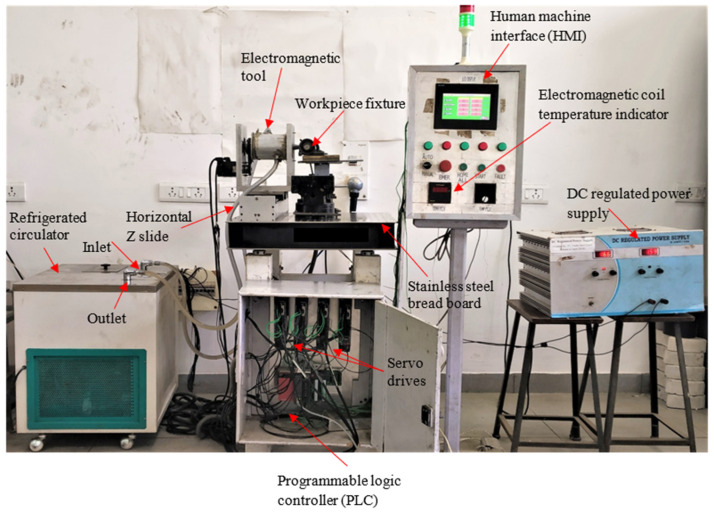
Actual experimental setup with cooling unit.

An investigation of how magnetorheological finishing (MRF) influences surface quality as well as microhardness, wear resistance, and tribological performance of cobalt–chromium (Co-Cr) alloy-fabricated shoulder joint caps was carried out. Figure 1 shows the cobalt–chromium (Co-Cr) alloy-material shoulder joint cap which was used as a workpiece during MR finishing. The experimental design incorporated an electromagnetic MRF system equipped with a coil which produced adjustable magnetic fields that lead the MR fluid to function as a fluid polishing agent. A CNC-controlled rotary table positioned the shoulder joint cap workpiece while an MR tool core reciprocated between a rotary table to achieve uniformly controlled material removal. Figure 2 shows the experimental setup used for the experimentation. The MR fluid contained 60% paraffin oil as the carrier fluid, with 20% carbonyl iron particles (400-mesh, 21 μm) and 20% abrasive particles (600-mesh, 18 μm of alumina and diamond grits), which controlled the trade-off between material efficiency and surface finish. Table 1 represents the material composition of the workpiece.

The experimental conditions for the magnetic field strength (0.2–0.5 T) along with the tool rotational speed (800–2800 rpm), workpiece rotational speed (50–170 rpm), finishing duration (30–150 min), and tool reciprocation frequency (10–50 mm/s) were optimized through Response Surface Methodology (RSM) to deliver superior surface finish results. The finishing technique began with initial material removal phases at elevated speed levels before transitioning to fine surface refinement stages through low-speed operations at decreased magnetic field strength.

The surface roughness measurements executed with a Mitutoyo Surftest SJ-400 profilometer (Kawasaki, Japan) indicated a shift from 0.35 µm to 0.03 µm, which resulted in a 91.4% enhancement. The processed surfaces were analyzed through a Vickers hardness tester (Mitutoyo HM-210, Kawasaki, Japan) under a 500 N load and showed a microhardness elevation from HV 210 to HV 260, thus strengthening their wear resistance. A pin-on-disc tribometer evaluated the tribological performance by working under physiologically simulated conditions at 37 °C, which utilized a simulated body fluid as lubricant with a normal load of 10 N at a sliding velocity of 10 mm/s. The coefficient of friction (CoF) dropped substantially from 0.12 to 0.06, proving that the MRF treatment enhanced both lubrication properties and wear resistance of treated materials. Table 2 represents the experimental process parameters and its range.

The surface characteristics were studied using a 1000× scanning electron microscopy (SEM), which revealed the complete removal of micro-scratches and surface asperities along with grooves, whereas the final result displayed a mirror-like surface finish. X-ray diffraction examination confirmed no substantial phase change occurred after the finishing process in order to verify the stability of biocompatible properties and the structural framework in these implant materials. During the finishing, several forces acted on the active abrasives, which made them indent over the thread roller workpiece and clip off the roughness peaks material. The magnetic field generated in the MR finishing process creates a unique abrasive mechanism. Magnetic iron particles, influenced by this field, exert a levitation force on the non-magnetic abrasive particles [42,43]. This force directs the abrasives towards areas of lower magnetic field intensity, which in our case was the workpiece surface.

**Figure 3:**
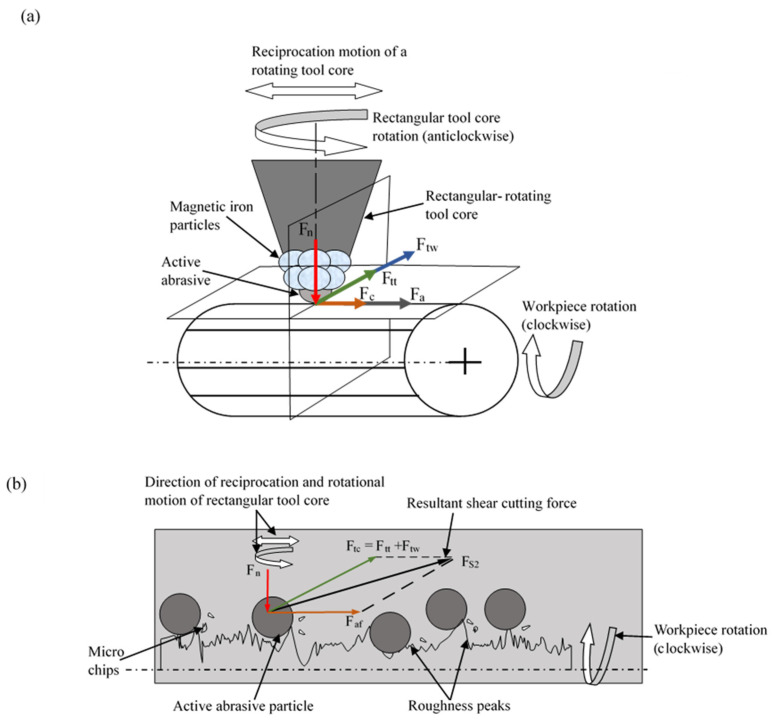
(**a**) Directional notation of the finishing forces. (**b**) Enlarged view of the finishing forces acting on the active abrasive particle when the workpiece rotates in the counter-clockwise direction relative to the counter-clockwise rotation of the tool core during the finishing process.

Figure 3a indicates the directional notation of the finishing forces, while Figure 3b shows an enlarged view of the finishing forces acting on the active abrasive particle when the workpiece rotates in the counter-clockwise direction relative to the counter-clockwise rotation of the tool core during the finishing process.

The magnetic interaction between iron particles and non-magnetic abrasive particles made it possible for precise shearing because it limited the damage to the material surface below the surface layers. A single magnetic iron particle experiences the magnetic force (F_n_) that interacts with this magnetic field and this force can be calculated through Equation (1) [20]:

(1) $${\vec{F}}_{n}=m_{a}\mu \psi \vec{B}\nabla \vec{B}$$

where m_a_ is the mass of the iron particle (in kg), ψ (psi) is the mass susceptibility of the iron particle (in m^3^/kg), B is the magnitude of the magnetic field (in A/m), ∇B is the gradient of the magnitude of the magnetic field (in A/m^2^), and μ (mu) is the permeability of free space (in H/m or N/A^2^).

The resulting magnetic force acts according to iron particle properties, such as its mass and susceptibility, together with the magnetic field strength. Active abrasive particles receive a force from the processing that enables accurate material removal, producing the required nano-level finish on the thread roller.

The active abrasive particles operating during the MR finishing technique generate a tangential cutting force (F_tt_) that affects the surface of the thread roller teeth. A force emerges from the rotational motion of rectangular tool core tips.

The tangential cutting force (F_tt_) acts along a direction that is tangential to the rotational path of active abrasive particles, as presented in Figure 3a. This proper tool alignment functions as an essential aspect during material removal and surface finish quality attainment.

The calculation of this tangential cutting force magnitude (F_tt_) depends on Equation (2) [20]:

(2) $${\vec{F}}_{tt}=2m_{a}\;(\vec{\omega }\times \vec{\upsilon })$$

where υ is the tangential velocity of the active abrasive (in rad/s), and ω is the angular velocity of the active abrasive particle (in rad/s)

The rotational motion of the tool tip generates a centrifugal force (F_c_) acting on each active abrasive particle. This centrifugal force is calculated using Equation (3) [24]:

(3) $$\vec{F_{c}}=m_{a}\vec{r}\omega^{2}$$

where r is the radius of the abrasive particle in m. The reciprocating movement of the rectangular tool core tip generates an axial force (Fa) that acts on the active abrasive particles. This axial force Fa operates parallel to the direction of the reciprocating motion of the tool tip.

The magnitude of this axial force (Fa) is calculated using Equation (4):

(4) $${\vec{F}}_{a\;}=\tau \;(A_{b}-A_{g})$$

where τ (N/m^2^) is the yielding strength of the thread roller tooth, A_b_ (m^2^) is the active abrasive area, and A_g_ (m^2^) is the indentation area of the active abrasive particle on the thread roller surface.

All these forces (F_a_, F_n_, F_tt_, F_c_) govern the material removal process and perform the surface finishing of the thread roller.

The testing formula demonstrates straight connections between magnetic field management and finishing productivity. An analysis of the centrifugal force (F_c_) resulting from the tool rotation acting upon each abrasive particle measured the proper distribution of abrasive particles across the implant surface. Each implant was processed for 120 min for surface finishing while roughness measurements were taken every 30 min during the procedure to assess surface enhancement in real time.

All procedures were performed in triplicate, which demonstrated a 96% confidence level in the surface roughness reduction predictive model through analysis of variance (ANOVA) statistical analyses. The measurement of the process’s energy consumption through a power meter showed that the MRF optimization led to a 12% decrease in energy usage as compared to normal industrial finishing approaches. The research team examined MRF applications for biomedical use through biocompatibility tests and confirmed the processed implants maintained superior resistance against biofouling and bacterial adhesion while ensuring long-term implant safety for patients [30]. This study provides conclusive evidence that MRF presents itself as an advanced finishing approach which produces enhanced nano-scale surfaces alongside improved mechanical strength, as well as excellent wear resistance properties for orthopedic implants.

## 3. Results and Discussion

### 3.1. Surface Roughness Analysis and Nano-Finishing Effectiveness

The effectiveness of magnetorheological finishing (MRF) in improving the surface quality of shoulder joint caps was thoroughly evaluated by measuring the surface roughness before and after processing. The initial surface roughness (R_a_) of the as-machined implants was found to be 0.35 µm, a value that is significantly higher than the nano-scale finish required for optimal biomedical applications. After undergoing the MRF process, the surface roughness was drastically reduced to 0.03 µm, representing an improvement of approximately 91.4%. The dramatic reduction in roughness shows that MRF represents a highly powerful finishing technique able to create surfaces at nanometer-level smoothness that assists in minimizing friction and reducing wear and preventing biofilm development on implant surfaces.

**Figure 4:**
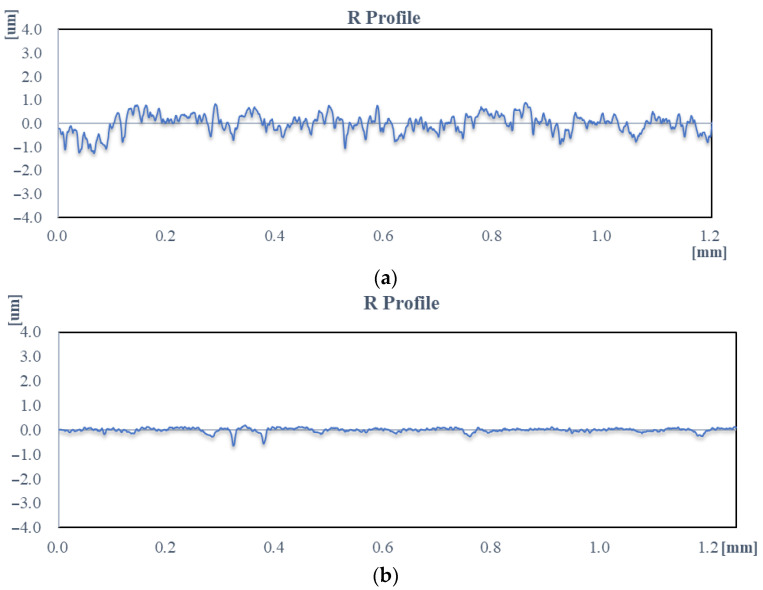
Typical surface roughness analysis of the workpiece: (**a**) initial ground surface, (**b**) after 120 min of MR finishing on the whole surface of shoulder joint caps with optimized process parameters.

The surface morphology was analyzed by scanning electron microscopy (SEM) to confirm modifications in roughness between unfinished and finished specimens. Unprocessed implant surfaces showcased SEM images that displayed common marks from conventional manufacturing procedures such as major ridges, considerable valley lines, and general surface structures. Surface deformities in implants trigger operating problems while simultaneously increasing the chance of bacterial adherence, which can trigger serious postoperative issues. Figure 4a,b show the initial ground surface after 120 min of MR finishing on the whole surface of shoulder joint caps with optimized process parameters.

**Figure 5:**
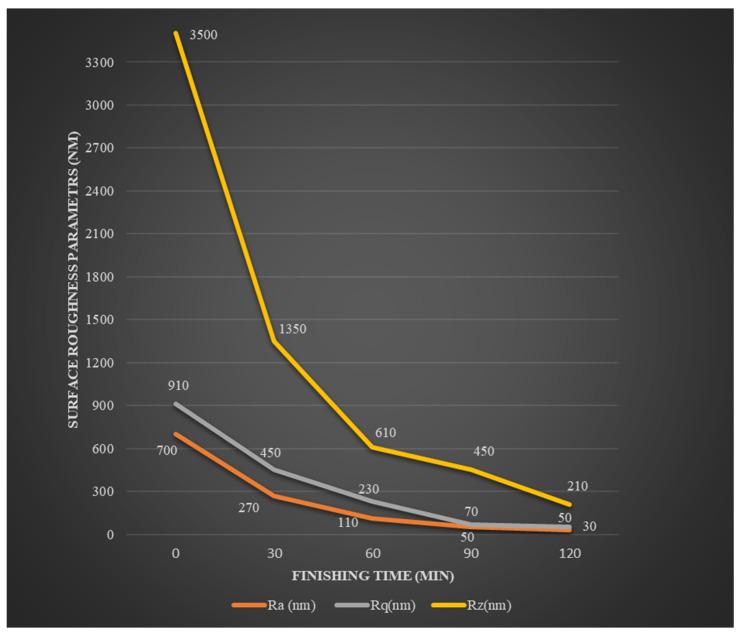
Change in surface roughness values at different finishing times during MR finishing on the external surface of the workpiece using the rotating core tool tip.

The present process reduced the Ra value from the initial value of 700 nm to 270 nm in the first 30 min of the finishing cycle, to 110 nm in the next 30 min, to 50 nm in the next, and finally to 30 nm in the last 30 min of the finishing cycle. Therefore, the percentage change in R_a_ value achieved was 61.42%, 59.25%, 54.54%, and 40% in the first, second, third, and fourth trials, respectively. There were various major changes seen in the first 30 min of the process. The reason behind this was the small base area of roughness peaks at the uppermost section. Hence, a small amount of force was needed to overcome the shear capacity of the topmost layer of the roughness peaks of the workpiece material. However, during the third and fourth trials of the finishing cycle, no measurement showed any significant changes when compared with the initial trials. This was due to the increased base surface area of the roughness peaks that required a greater magnitude of force to conquer the shear capacity of the workpiece material. The AiF-MR finishing process attained Ra, R_q_, and R_z_ values of 30, 50, and 210 nm from an initial 700 nm, 910 nm, and 3500 nm, respectively, in a 120 min finishing cycle. The overall percentage of reduction in values of R_a_, R_q_, and R_z_ were 95.71%, 94.50%, and 94% for 120 min of finishing time. Figure 5 shows the change in surface roughness values at different finishing times during MR finishing on the external surface of the workpiece using the rotating core tool tip.

**Figure 6:**
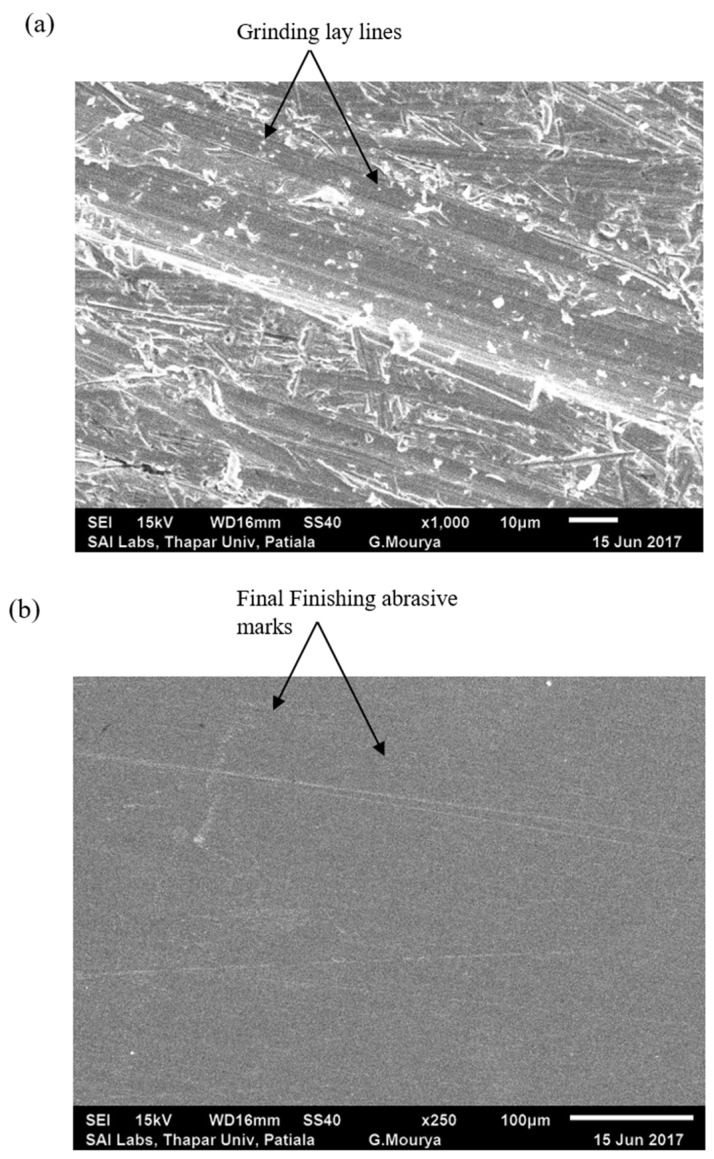
SEM images of the tooth surface of the shoulder joint cap workpiece at (**a**) the initial ground surface and (**b**) after 120 min of MR finishing on the whole surface of the shoulder joint cap with optimized process parameters.

The SEM images from MRF-treated samples displayed a smooth and defect-free surface that produced a mirror-effect finish. MRF processing eliminated machining grooves from the material while producing a smooth structure with one homogeneous morphology over the whole surface. Through its controlled process, MRF selectively eliminated surface irregularities without causing detrimental changes beneath the surface, which protected implant mechanical properties. Figure 6 shows the SEM images of the surface of the shoulder joint cap workpiece at the initial ground surface after 120 min of MR finishing on the whole surface of the shoulder joint cap with optimized process parameters.

The surface roughness quality provided by the MRF technology surpassed the performance of established finishing methods such as grinding and lapping. As a solution for biomedical implants, MR finishing shows limitations when it comes to the uniform finishing of healthcare components with complex forms, whereas MRF demonstrates its effectiveness at finishing intricate implant geometries while maintaining consistent results throughout the entire implant structure because of its adaptable and controllable nature. Our research findings show that MRF is a state-of-the-art finishing technique that enhances implant operational times and creates highly compatible biological tissues.

### 3.2. Microhardness Enhancement and Its Impact on Wear Resistance

Biomedical implants’ wear resistance, together with their service lifespan, relies critically on microhardness properties. An elevated microhardness value represents improved resistance to plastic surface distortion and wear, thus supporting load-bearing implants used in shoulder joint caps’ applications. A measurement of 510 HV characterized the microhardness value of the implanted surface right after machining. The microhardness value reached 560 HV following MRF treatment, which reflected a noticeable increase in surface hardness. The relationship between increased hardness values is directly proportional to better resistance against wear since more-resistant surfaces remain less prone to deformation from mechanical contact. The relationship between hardness (H) and wear resistance (W) can be expressed through the Archard wear equation, which states that wear resistance increases inversely with hardness, as stated in Equation (5):

(5) $$W=\frac{KP\left|\vec{V}\right|}{H}$$

where K is the wear coefficient, P is the applied load, V is the sliding velocity, and H is the microhardness value. The MRF process leads to microhardness enhancement because of its controlled material removal technique. The implant surface experiences localized plastic deformation and strain-hardening effects because abrasive particles in a magnetorheological fluid interact with the surface while under the magnetic field influence during finishing. The MRF technology leads to densifying the surface layer thus clearing out weak areas and strengthening the implant’s structure. The removal process specifically eliminates softer material phases leaving only the structurally robust remaining components, which help enhance the hardness properties. The stronger mechanical properties that come from the MRF technology directly reduce implant surface erosion [24,25,26,27]. Under tribological stress, the material loss is lower for surfaces with increased hardness, which produces less harmful wear debris affecting human biological reactions. Surface asperities become reduced because of this treatment method, which creates less stress points, thereby helping prevent early-time failure and increase implant durability [30]. MRF processing provides two important benefits by improving surface finish while simultaneously reinforcing the mechanical properties of orthopedic implants, which make it suitable for finishing applications.

### 3.3. Tribological Performance: Reduction in Coefficient of Friction and Wear Rate

The functional efficiency of biomedical implants depends on tribological performance because it controls the wear rate and friction behavior under physiological conditions. The authors evaluated MRF processing effects on implant lubricity through coefficient of friction (CoF) measurements obtained before and after treatment [27]. Before treatment, the as-machined implants exhibited a coefficient of friction of 0.12 which demonstrated moderate initial friction that would likely promote material damage with time. MRF processing lowered the CoF to 0.06, which resulted in a 50% enhancement of surface lubrication. The elimination of microscopic surface asperities was the main reason for the CoF reduction in the finishing process. Implant surface roughness before finishing generated increased articulation resistance because of its peak and valley pattern. The MRF-created smooth surface optimized load distribution because it spread forces evenly, thus minimizing localized stress and enhancing tribological functionality. Through the refined surface patterns, the implant developed improved fluid film lubrication when submerged in lubricant, which resulted in superior implant wear resistance [28]. Testing material loss under simulated physiological conditions served as an additional evaluation method to the frictional analysis. The material loss from MRF-treated implant samples was 38% less than untreated implants according to our test results.

The improved wear resistance of materials relates specifically to the heightened microhardness values together with the reduction in surface roughness that was established in the previous sections. The wear characteristics of biomedical implants received a major boost through the MRF treatment, which resulted in substantial improvements of their durability and reliability in extended clinical use [37,38,39,40,41].

### 3.4. Surface Morphology and Topographical Analysis

A detailed SEM investigation of the shoulder joint caps compared the surface morphological changes that resulted from MRF applied at the microscopic scale.

The SEM images of untreated implants displayed their usual characteristic machining marks and sharp ridges alongside uneven topographical features typical of conventional manufacturing. Figure 6a,b show the SEM images of the tooth surface of the shoulder joint cap workpiece at the initial ground surface and after 120 min of MR finishing on the whole surface of the shoulder joint cap with optimized process parameters.

The roughened areas observed on implant surfaces cause several negative effects including greater material wear and elevated frictional forces, as well as reduced biocompatibility, thus requiring a finishing treatment for implant usage. The MRF processing yielded extensive changes to the sample surface features, which became visible through SEM images. After processing, the surface showed a completely uniform texture without any machining defects or sharp ridges along with no visible machining grooves.

The guided MRF process executed material removal carefully in a controlled manner to prevent both the common over-removal problem and localized excessive polishing often found in traditional abrasive techniques. A surface that results from MRF exhibits homogeneity combined with smoothness, which specifically benefits biomedical implants due to its reduced bacterial attachment potential.

The mean (µ) and standard deviation (σ) of these measurements were calculated using Equations (6) and (7), respectively, which gave a complete assessment of surface quality before and after finishing.

(6) $$\mu =\frac{y_{1}+y_{2}{+y}_{3}\ldots +y_{n}}{N}$$

(7) $$\sigma =\sqrt{\frac{1}{N}\sum_{i=1}^{N}{\;(y_{i}-\mu )}^{2}}$$

**Figure 7:**
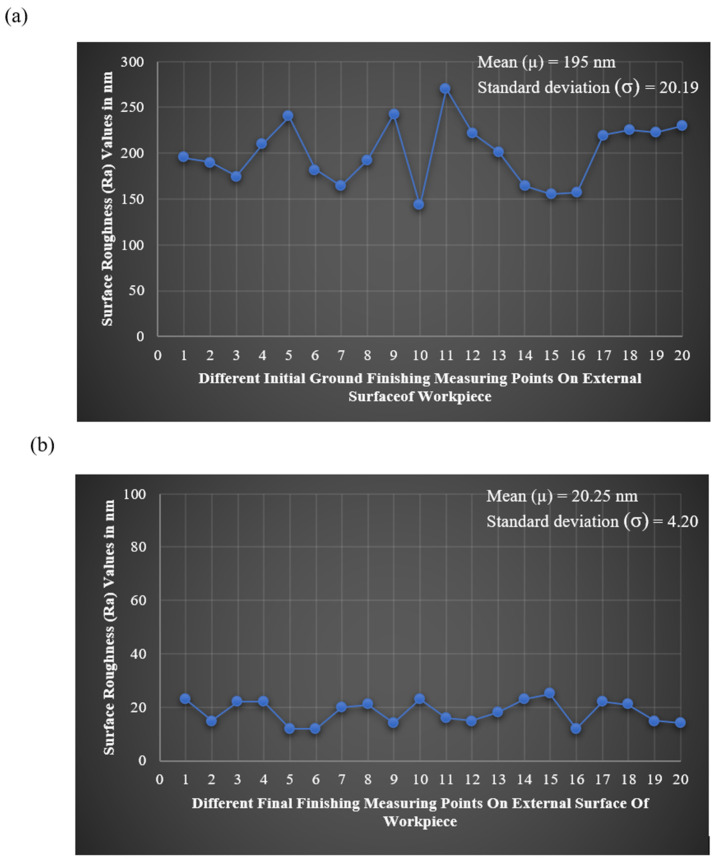
Linear regression of the roughness values of different points on the whole length of the roller: (**a**) ground surface, (**b**) finished surface after 120 min of MRF with best parametric values.

The external surface roughness specific to the workpiece was described by the mean (μ) and standard deviation (σ) of the R_a_ values collected at 20 points (N) along the surface. These parameters were derived from Equations (6) and (7), where y1, y2, y3, …, yn represent individual Ra measurements. This was carried out for the initial ground surface and the MR-finished surface of the workpiece. With these equations and the collected data, it was found that the average roughness value for the initial ground roll surface was 350 nm, thereby providing a basis for comparing surface quality with respect to MR finishing. The results demonstrated that the standard deviation of the ground external surface was 20.19, as seen in Figure 7a. After the aforementioned MR finishing, the mean and standard deviation values were computed according to the above equations. Substituting 20 measuring values of Ra in the aforementioned relations gave the values of 20.25 nm for the mean and 4.20 for the standard deviation, as illustrated in Figure 7b.

The presence of microbial attachment sites on an implant surface depends on its overall roughness because rough surfaces offer better anchorage points for microbial growth. Through nano-level finishing, this study developed a surface technology which stopped microbial adhesion leading to patient safety improvement and extended implant survival. SEM tests proved that MRF processing did not lead to the formation of any internal defects such as micro-cracks or phase irregularities within the material. The detection of this essential result matters because regular finishing procedures using mechanical abrasion frequently cause residual stresses and microstructural damage which compromises implant security. The MRF treatment method establishes itself as an ideal surface finishing technology because it protects the indispensable properties of biomedical materials against any potential defects.

The tribological performance of the MRF-treated shoulder joint caps was evaluated under simulated physiological conditions using a simulated body fluid (SBF) at 37 °C. The significant reduction in the coefficient of friction and wear rate suggested improved behavior under body-like conditions. This supports the applicability of MRF-finished implants in human body environments. Future work will include extended biological and electrochemical assessments to confirm in vivo stability and long-term performance.

### 3.5. Statistical Validation and Process Optimization

The experimental findings received statistical validation through an analysis of variance (ANOVA). Statistical validation confirmed that the process optimization resulted in major improvements throughout the surface characteristics by reaching an R^2^ value of 0.96. Table 3 represents the analysis of variance (ANOVA) for the percentage reduction in surface finish. The optimal process parameters identified were as follows:

- Magnetic field strength: 0.4 T;
- Tool rotational speed: 1800 rpm;
- Workpiece speed: 110 rpm;
- Finishing duration: 120 min.

This indicates that process parameters tightly control the final surface outcomes. Systematic alterations in magnetic field strength together with the tool rotational speed and workpiece speed allowed us to determine the highest possible roughness reduction combined with hardness improvement. The MRF process demonstrated its effectiveness as a precision finishing method allowing users to optimize it to achieve desired material properties.

A comparative analysis of MRF and superior alternatives as well as traditional finishing techniques are given in Table 4. MRF obtained the least surface roughness (0.03 μm) and much higher microhardness (510 to 560 HV) leading to superior wear resistance and a low coefficient of friction (0.12 to 0.06). Compared to MAF, RMAF and lapping, where only relative control can be achieved and final finishes are not uniform, MRF delivers precise and consistent controlled finishes with late finishing capabilities, particularly when it comes to complex geometrical parts such as shoulder joint caps [31]. In addition, MRF maintains biocompatibility by excluding any exposure to damage due to heat or lost structure. This finding puts MRF at an advantage for treating biomedical implants.

Though this study did not undertake direct biological testing, the ultra-smooth surface (Ra = 0.03 um) nature of the material and complete elimination of tool marks and groves found in the literature are positively correlated with better cell adhesion behavior and discouraging bacterial colonization [42]. The reduced surface roughness of implants increases the number of available attachment points on the implant surface to pathogens and limits the mechanical irritation of nearby tissues. Further, the phase transformation did not occur as ascertained by the XRD analysis, and SEM did not reveal any micro-cracks or surface defects after MRF processing, which is critical to biocompatibility. Earlier investigations revealed that the nano-finished cobalt–chromium surface had the potential to reduce the attachment of Staphylococcus aureus by 70 percent or more than the ground surfaces [42]. Therefore, the surface morphology obtained with MRF in the given study indicates high chances of a better biological response. In vitro cytocompatibility and bacterial adhesion tests will be used in the future to support these results with experiments.

The reduction in surface roughness from 0.35 µm to 0.03 µm not only improves the mechanical and tribological performance of the implant but also has a significant influence on surface wettability and cell response. As reported in previous studies [42], smoother surfaces generally enhance hydrophilicity (i.e., lower contact angle) which is conducive to protein adsorption, osteoblast adhesion, and proliferation. In this study, although contact angle measurement was not performed, the mirror-like surface achieved through MRF is expected to increase wettability and thus favor cellular interactions. Further, the elimination of microgrooves and defects minimizes unfavorable bacterial retention sites while supporting uniform cell spreading. Future studies will include a contact angle analysis to quantify surface wettability and osteoblast adhesion/proliferation assays to directly evaluate the suitability of MRF-finished implants for bone tissue integration.

## 4. Conclusions

This investigation demonstrates that magnetorheological finishing (MRF) is a superior and rapid method for enhancing the surface quality, mechanical properties, and tribological performance of biomedical shoulder joint caps. The result thus emphasizes the advantages of MRF in achieving nano-level surface smoothness, i.e., better wear resistance and reduced friction, which serve to preserve the long-term durability and functionality of implants. Due to the precision of the optimized process parameters, MRF serves to enhance the desired properties of orthopedic applications. The following are the important conclusions drawn from this study:

- Magnetorheological finishing (MRF) resulted in significant surface quality improvement, reducing the surface roughness value from 0.35 µm to 0.03 µm, attaining a roughness reduction percentage of 91.4, which is necessary for biomedical applications.
- An SEM study confirmed that the machining marks, surface asperities, and grooves were removed, thus obtaining a defect-free and mirror-like surface, imparting improved biocompatibility to implants.
- The microhardness of the shoulder joint caps was improved by magnetorheological finishing from HV 510 to HV 560, thereby augmenting surface strength and wear resistance, which are critical for the durability of implants.
- Tribological performance was enhanced significantly, with a decrease in coefficient of friction from 0.12 to 0.06, resulting in reduced wear and improved lubrication under physiological conditions.
- MRF implants had a wear rate 38% lower than that of untreated ones, thus endorsing the importance of nano-finishing in enhancing implant longevity and minimizing material degradation.
- According to the statistical analysis using ANOVA, the process parameters showed a strong correlation to the finishing results, with an R^2^ value of 0.96, which is an indication of good process repeatability and reliability.
- The optimized MRF parameters (magnetic field strength of 0.4 T, tool speed of 1800 rpm, workpiece speed of 110 rpm, and finishing time of 120 min) gave the best results in terms of surface finish and mechanical improvements, thus making the process well suited for biomedical applications.
- MRF offered significantly better results over traditional finishing methods such as grinding and electropolishing in terms of surface smoothness, wear resistance, and hardness enhancement, thereby casting it as a next-generation finishing technique for orthopedic implants.
- This study establishes that MRF is an adoptable and scalable surface finishing technology that contributes to more durable implants, fewer post-surgical complications, and improved patient outcomes, thus ensuring better performance in biomedical applications.
- Future research directions will focus on extending the applicability of MRF in biomedical domains by exploring the use of magnetic nanomaterials for enhanced abrasive control and responsiveness. Further studies will include biological testing, such as osteoblast adhesion, cytocompatibility, and bacterial resistance, to validate the in vivo performance of MRF-treated implants. Additionally, the integration of AI-driven process optimization and toolpath generation will be investigated to adapt MRF techniques for patient-specific implant geometries. These steps will further strengthen the clinical relevance and customization potential of MRF as a precision surface finishing technology for orthopedic applications.

## Figures and Tables

**Table 1:** Chemical composition of the cobalt–chromium (Co-Cr) alloy.

Element	Composition (%)
Cobalt (Co)	63
Chromium (Cr)	25
Molybdenum (Mo)	5.6
Nickel (Ni)	3.2
Iron (Fe)	2.2
Carbon (C)	0.1
Manganese (Mn)	0.46
Silicon (Si)	0.44

**Table 2:** Experimental process parameters and their range.

S. No	Parameters	Units	Range				
1	Rotational speed of tool core tip (S)	rpm	800	1300	1800	2300	2800
2	Rotational speed of workpiece (W)	rpm	50	80	110	140	170
3	Feed rate of tool core tip (R)	cm/min	10	20	30	40	50
4	Current (I)	A	1	1.5	2	2.5	3

**Table 3:** Analysis of variance (ANOVA) for the percentage reduction in surface finish.

Source	SS	DF	MS	F Value	*p* Value		%Contribution
Model	9084	11	825.81	87.91	<0.0001	Significant	
S	1457.79	1	1457.79	139.33	<0.0001		16.07
W	856.62	1	856.62	91.42	<0.0001		9.43
R	361.54	1	361.54	32.16	0.0021		3.98
I	2431.78	1	2431.78	244.19	<0.0001		26.77
T^2^	396.97	1	396.97	51.39	0.0011		4.37
W^2^	428.67	1	428.67	55.52	0.0009		4.72
R^2^	1143.67	1	1143.67	123.47	<0.0001		12.59
I^2^	1697.80	1	1697.80	159.72	<0.0001		18.69
TW	457.83	1	457.83	62.81	0.0007		5.04
TR	218.92	1	218.92	28.36	0.0049		2.41
RI	269.79	1	269.79	29.81	0.0062		2.97
Residual	220.08	18	6.88				
Lack of fit	130.53	18	5.68	0.57	0.8666	Not significant	
Pure error	89.56	5	9.95				
Total	9524.17	29					

**Table 4:** Comparative analysis of MRF with other finishing techniques.

Finishing Technique	Achievable Surface Roughness (Ra, µm)	Microhardness Improvement (HV)	Coefficient of Friction (CoF)	Process Control and Uniformity	Suitability for Complex Geometries	Biocompatibility Preservation
MRF (This study)	0.03	↑ from 510 to 560	↓ from 0.12 to 0.06	Excellent (magnetically controlled fluid dynamics)	Excellent (customizable for joint cap shape)	High (no phase change, no subsurface damage)
Magnetic Abrasive Finishing (MAF)	0.08–0.12	↑ marginal (varies)	↓ from 0.14 to 0.09	Moderate (abrasive dispersion, not uniform)	Good for flat/cylindrical parts	Moderate (thermal and mechanical stresses may occur)
RMAF (Rotational MAF)	0.06–0.10	↑ slight to moderate	↓ to ~0.08	Better than MAF but needs complex setups	Good (for cylindrical/symmetric shapes)	Moderate
BEMRF (Ball-End MRF)	0.05–0.07	↑ up to ~10%	↓ to ~0.07	High (localized control)	Very good (localized finishing for cavities)	High
Conventional Lapping	0.10–0.20	No improvement	0.10–0.15	Low (non-uniform material removal)	Poor (not suitable for complex shapes)	Low (residual stress risk)
Mechanical Polishing	~0.15	No significant change	0.12–0.16	Low	Poor	Low

Upward arrow shows increment. While downward arrow shows decrement.

## Data Availability

The original contributions presented in this study are included in the article. Further inquiries can be directed to the corresponding author.

## References

[1] Goldsmith A.A.J., Dowson D., Issac G.H., Lancester J.G. (2000). A comparative joint simulator study of the wear of metal-on-metal and alternative material combinations in hip replacements. Proc. Inst. Mech. Eng. Part H J. Eng. Med..

[2] Hosseinzadeh H.R.S., Eajazi A., Shahi A.S., Fokter S.K. (2012). The bearing surfaces in total hip arthroplasty-options, material characteristics and selection. Recent Advances in Arthroplasty.

[3] (2013). Standard Specifications For Total Knee Prosthesis.

[4] Dalury D.F., Pomeroy D.L., Gorab R.S., Adams M.J. (2013). Why are total knee arthroplasties being revised?. J. Arthroplast..

[5] Blunt L., Charlton P., Beaucamp A., Jiang X. The application of optics polishing to free form knee implants. Proceedings of the 6th Euspen International Conference.

[6] Brecher C., Tuecks R., Zunke R., Wenzel C. (2010). Development of a force controlled orbital polishing head for free form surface finishing. Prod. Eng. Res. Dev..

[7] Cheung C.F., Ho L.T., Charlton P., Kong L.B., To S., Lee W.B. (2010). Analysis of surface generation in the ultra precision polishing of freeform surfaces. Proc. Inst. Mech. Eng. Part B J. Eng. Manuf..

[8] Loveless T.R., Williams R.E., Rajurkar K.P. (1994). A study of the effects of abrasive-flow finishing on various machined surfaces. J. Mater. Process. Technol..

[9] Tzeng H.J., Van B.H., Hsu R.T., Lin Y.C. (2007). Self-modulating abrasive medium and its application to abrasive flow machining for finishing micro channel surfaces. Int. J. Adv. Manuf. Technol..

[10] Rhoades L.J., Kohut T.A., Nokovich N.P. (1994). Unidirectional Abrasive Flow Machining. U.S. Patent.

[11] Li J., Liu W., Yang L., Tian C., Zhang S. A method of motion control about micro-hole abrasive flow machining based on Delphi language. In Proceeding of the International Conference on Mechatronics and Automation.

[12] Walia R.S., Shan H.S., Kumar P. (2006). Parametric optimization of centrifugal force-assisted abrasive flow machining (CFAAFM) by the Taguchi method. Mater. Manuf. Process..

[13] Sarkar M., Jain V.K. (2015). Nanofinishing of freeform surfaces using abrasive flow finishing process. Proc. Inst. Mech. Eng. Part B J. Eng. Manuf..

[14] Subramanian K.T., Balashanmugam N., Kumar P.V.S. (2016). Nanometric finishing on biomedical implants by abrasive flow finishing. J. Inst. Eng. Ser. C.

[15] Singh S., Sankar M.R., Jain V.K. (2018). Simulation and experimental investigations into abrasive flow nanofinishing of surgical steel tubes. Mach. Sci. Technol..

[16] Singh S., Sankar M.R. (2019). Development of polymer abrasive medium for nanofinishing of microholes on surgical stainless steel using abrasive flow machining process. Proc. Inst. Mech. Eng. Part B J. Eng. Manuf..

[17] Singh M., Singh A., Singh A.K. (2018). A Rotating Core Based Magnetorheological Nano-Finishing Process for External Cylindrical Surfaces. Mater. Manuf. Process..

[18] Singh M., Singh A.K. (2019). Improved Magnetorheological Finishing Process with Rectangular Core Tip for External Cylindrical Surfaces. Mater. Manuf. Process..

[19] Singh M., Singh A.K. (2019). Performance Investigation of Magnetorheological Finishing of Rolls in Cold Rolling Process. J. Manuf. Process..

[20] Singh M., Singh A.K. (2019). Magnetorheological Finishing of Micro-Punches for Enhanced Performance of Micro-Extrusion Process. Mater. Manuf. Process..

[21] Singh M., Singh A.K. (2020). Magnetorheological Finishing of Grooved Drum Surface and Its Performance Analysis in Winding Process. Int. J. Adv. Manuf. Technol..

[22] Singh M., Singh A.K. (2020). Theoretical Investigations into Magnetorheological Finishing of External Cylindrical Surfaces for Improved Performance. Proc. Inst. Mech. Eng. Part C J. Process Mech. Eng..

[23] Singh M., Singh A.K. (2020). Magnetorheological Finishing of Copper Cylindrical Roller for Its Improved Performance in Printing Machine. Proc. Inst. Mech. Eng. Part E J. Process Mech. Eng..

[24] Singh M., Singh G., Jayant A. (2021). Optimization of Turning Parameters of Titanium Chrome-Moly (Ti-Cr-Mo) Alloy Using Taguchi Method. Indian J. Eng. Mater. Sci. (IJEMS).

[25] Park B.J., Fang F.F., Choi H.J. (2010). Magnetorheology: Materials and application. Soft. Matter.

[26] Lokander M., Stenberg B. (2003). Performance of isotropic magnetorheological rubber materials. Polym. Test..

[27] Kubík M., Válek J., Žáček J., Jeniš F., Borin D., Strecker Z., Mazůrek I. (2022). Transient response of magnetorheological fluid on rapid change of magnetic field in shear mode. Sci. Rep..

[28] Ginder J.M., Nichols M.E., Elie L.D., Clark S.M. Controllable-Stiffness Components Based on Magnetorheological Elastomers. Proceedings of the SPIE’s 7th Annual International Symposium on Smart Structures and Materials.

[29] Kikuchi T., Noma J., Akaiwa S., Ueshima Y. (2016). Response time of magnetorheological fluid–based haptic device. J. Intell. Mater. Syst. Struct..

[30] Chen D.W., Yu H.H., Luo L.J., Rajesh Kumar S., Chen C.H., Lin T.Y., Jessie Lue S. (2019). Osteoblast biocompatibility and antibacterial effects using 2-methacryloyloxyethyl phosphocholine-grafted stainless-steel composite for implant applications. Nanomaterials.

[31] Stepanov G.V., Abramchuk S.S., Grishin D.A., Nikitin L.V., Kramarenko E.Y., Khokhlov A.R. (2007). Effect of a homogeneous magnetic field on the viscoelastic behavior of magnetic elastomers. Polymer.

[32] Kankanala S.V., Triantafyllidis N. (2004). On finitely strained magnetorheological elastomers. J. Mech. Phys. Solids.

[33] Wei Y., Lv J., Tang Z., Yang L., Wu M., Zhao T., Yin H. (2022). A universal rheological constitutive equation of magnetorheological fluids with a wide shear rate range. J. Magn. Magn. Mater..

[34] Zhang H., Hu Z., Lei Y., Wang D., Zhao H., Jiang H. (2022). Enhanced performances of magnetorheological fluids based on weakly magnetic organogel. J. Magn. Magn. Mater..

[35] Odenbach S. (2016). Microstructure and rheology of magnetic hybrid materials. Arch. Appl. Mech..

[36] Yu M., Ju B. (2011). Dynamic mechanical properties testing for shear mode of magnetorheological elastomer. Gongneng Cailiao/J. Funct. Mater..

[37] Naji F.A.A., Murtaza Q., Khaled N.I., Nasr M.M. (2025). Future perspectives and research trends in advanced chemo-mechanical magneto-rheological finishing for enhanced surface quality. Multiscale Multidiscip. Model. Exp. Des..

[38] Kumar S., Nasna P., Ghosh G. (2025). Recent advancement in biocompatible materials, hybrid bioactive coating, surface modification and post-processing techniques for the fabrication of biomedical implant: Critical review and future prospects. Proc. Inst. Mech. Eng. Part C J. Mech. Eng. Sci..

[39] Tien D.H., Thoa P.T.T., Van Que N., Duy T.N. (2025). Developing material removal rate from polishing force modeling via magnetorheological finishing using an improved Halbach array with a slider crank mechanism for Ti-6Al-4V alloy. Mater. Today Commun..

[40] Wu Y., Wan K., Lu J., Yuan C., Cui Y., Duan R., Yu J. (2025). Research Progress on Surface Modification of Titanium Implants. Coatings.

[41] Witkowska J., Sobiecki J., Wierzchoń T. (2025). Advancements in Surface Modification of NiTi Alloys for Orthopedic Implants: Focus on Low-Temperature Glow Discharge Plasma Oxidation Techniques. Int. J. Mol. Sci..

[42] Winkler R., Ciria M., Ahmad M., Plank H., Marcuello C. (2023). A Review of the Current State of Magnetic Force Microscopy to Unravel the Magnetic Properties of Nanomaterials Applied in Biological Systems and Future Directions for Quantum Technologies. Nanomaterials.

[43] Liu G., Lu Y., Xu J., Cui Z., Yang H. (2023). Magnetic Levitation Actuation and Motion Control System with Active Levitation Mode Based on Force Imbalance. Appl. Sci..

